# Asthma and corticosteroids “*odi et amo”:* how to deal with it?

**DOI:** 10.3389/falgy.2026.1772380

**Published:** 2026-04-13

**Authors:** Luca Borsari, Laura Carbonieri, Luca M. Verdini, Valentina Ruggieri, Bianca Beghe’

**Affiliations:** 1 Department of Medical and Surgical Sciences, Section of Respiratory Diseases, University of Modena and Reggio Emilia, Modena, Italy; 2 Department of Life Sciences, University of Modena and Reggio Emilia, Modena, Italy

**Keywords:** adverse events, airway inflammation, biologics, corticosteroid stewardship, corticosteroids, inhaled, pharmacological strategies, screening

## Abstract

Asthma is a chronic, heterogeneous respiratory disease that affects more than 300 million individuals worldwide and is responsible for substantial morbidity, mortality and healthcare burden. Chronic airway inflammation plays a central role in symptom expression, disease progression, and response to therapy. Corticosteroids, both inhaled and systemic, are the cornerstone of asthma treatment. However, it is well known that the use and abuse of systemic corticosteroids is almost invariably associated to the development of significant acute and chronic adverse events. Epidemiological data report that despite the availability of new target therapy, e.g., biologics, in real practice systemic steroids are still overused, and in this review, we highlight strategies 1) to identify and screen patients at risk of developing corticosteroids adverse events and 2) to reduce them.

## Introduction

Asthma is a chronic, heterogeneous respiratory disease defined by a history of respiratory symptoms such as wheeze, shortness of breath, chest tightness, and cough, that vary over time, together with variable expiratory airflow limitation (1). Chronic airway inflammation plays a central role in symptom expression, disease progression, and response to therapy (2). The inflammatory process in asthma is complex, involving numerous immune cells and mediators responsible of the different endo-phenotypes of the disease. The most common endo-phenotype is Type 2 (T2)-high asthma, characterized by eosinophilic airway inflammation driven by cytokines such as interleukin (IL)-4, IL-5, and IL-13 (3, 4). T2-high asthma is typically responsive to corticosteroids (5, 6) and more recently to biologics (7, 8). By contrast, T2-low asthma is often neutrophilic or paucigranulocytic, less responsive to corticosteroids and biologics, remaining a major challenge in disease management (4, 9).

Corticosteroids (CS) have been the cornerstone of asthma treatment for almost 75 years (10). Since their introduction, systemic CS have provided effective control of asthma symptoms and exacerbations (1, 11). However, their use and often abuse quickly led to the development of significant adverse events (AEs) (12–16). The introduction in 1972 of inhaled corticosteroids (ICS) significantly reduced the use of systemic CS and the associated AEs without compromising clinical efficacy. From 2014 GINA guidelines started to consider systemic CS as a second choice maintenance treatment for patients with severe asthma not eligible for targeted therapies (17). Thanks to this change in guidelines, the increased awareness of the CS-related AEs and the availability of new biological therapies, the use of maintenance systemic CS in asthmatic patients declined (18–21), even if, their use is still too common (22, 23). Finally, there is an increasing evidence that even ICS-related AEs are not negligible (24). These observations highlight the urgent need for strategies to further minimize cumulative doses of CS to prevent their side effects (16).

The aims of this manuscript are: I) to report the use of CS in clinical practice and the related adverse events; II) to identify patients at risk of developing CS-related AEs; III) to report screening tests for steroid-related AEs; and, finally IV) to describe the available pharmacological strategies to reduce the burden of CS side-effects.

## Corticosteroids in asthma

Corticosteroids are the cornerstone of asthma management due to their potent anti-inflammatory effects (22). CS molecules diffuse across cell membranes, and act by binding the glucocorticoid receptor (GR). This regulates the transcription of genes encoding anti-inflammatory proteins and the repression of pro-inflammatory transcription factors (25, 26). Systemically administered CS are well absorbed orally, rapidly distributed to tissues, and extensively metabolized by the liver via CYP3A enzymes, even if renal metabolism also contributes (27). For the purpose of this review, we will refer to oral corticosteroids (OCS) as the primary route of systemic corticosteroid administration, although intravenous corticosteroids are also used particularly in the management of asthma exacerbations. OCS are classified according to potency and mineralocorticoid effects expressed relative to hydrocortisone, and duration of hypothalamic-pituitary-adrenal axis suppression. Structural modifications to the steroid molecule are designed to increase potency as well as to minimize mineralocorticoid effects (28). In asthmatic patients, the inhalation route is the preferred as it acts locally while minimizing the systemic side-effects (29). Upon inhalation, a considerable proportion of the administered dose of ICS is deposited in the oropharyngeal cavity—ranging from 40% to 90%—while only 10% to 60% reaches the lungs. If the oropharyngeal fraction is not rinsed out, it may be swallowed and absorbed via the gastrointestinal tract, contributing to systemic exposure (30). While the primary target of ICS are the airways, there is a pulmonary fraction, dependent of devices and mode of inhalation, that is absorbed directly across the alveolar-capillary membrane, and thus may cause systemic effects (31). The therapeutic index—the ratio between pulmonary efficacy and systemic adverse effects—varies considerably among ICS molecules depending on their pharmacokinetic and pharmacodynamic properties (29). Notably, GR occupancy studies have shown that >90% receptor saturation in the lungs can be achieved even at relatively low ICS doses, suggesting that the increase of ICS dose provides little additional benefit increasing the risk of systemic toxicity. Additionally, the duration of GR occupancy varies significantly across molecules. Among current ICS options, fluticasone furoate (FF) stands out for its exceptionally high GR-binding affinity, slow dissociation rate, and prolonged lung retention, all contributing to superior pulmonary potency and a wider therapeutic index. This molecule therefore offers a potential therapeutic advantage, particularly for patients with poor adherence to therapy. However, to date, FF in combination with vilanterol remains indicated in GINA Track 2, while budesonide or beclomethasone in combination with formoterol remain the first choice in GINA Track 1, despite both budesonide and beclomethasone having greater systemic effects and lower affinity for RBCs. This is due to the opportunity to use ICS/formoterol combinations as needed reducing the burden of inhaled steroids (32–34).

### Prevalence of OCS use

Although current guidelines suggest that maintenance treatment with OCS should be the very last option for patients with severe asthma, OCS are still frequently used in clinical practice. As expected, the burden of OCS use is more significant in patients with severe asthma and data from the International Severe Asthma Registry on almost 5,000 patients showed that >30% were on maintenance OCS (mOCS) while >50% were receiving several OCS bursts (35). Similar data have been reported in the Italian Registries of Severe Asthma (IRSA and SANI) where mOCS use ranges from 32% to 64% (36, 37). Moreover, in clinical practice the use of OCS bursts in patients with mild to moderate asthma are relatively common worldwide. Indeed, a Korean retrospective study amongst general asthma population reported that 27.7% were OCS users and 2.1% were on mOCS in a population with less than 20% of patients with severe asthma (GINA step 5) (38). Consistently with these data a Systematic Review by Bleecker et al. reported that OCS use was approximately 20%–48% of patients in the general asthma population. Finally, the use of long-term OCS was reported in 1.3% of patients with non-severe disease, while amongst patients with severe or uncontrolled asthma 20%–60% were reported to have received maintenance OCS (22).

### CS related adverse events

Acute and chronic adverse events (AEs) associated with OCS use have been extensively studied and can arise from both long-term treatment and short bursts. Acute and chronic AEs and their prevalence in patients with severe asthma are reported in Table 1.

**Table 1:** Acute and chronic adverse events of corticosteroids (CS).

Type of adverse event	Specific manifestations	Prevalence/Relative Risk
ACUTE	*Infections*	Pneumonia (28% vs. 11% in non-users); Opportunistic infections (1.5% vs. 0.4%) (22)
	*Gastrointestinal*	Dyspepsia (∼4%); Peptic ulcers; Short-term bursts (5–30 days) increase GI bleeding risk (IRR ≈ 1.4) (13, 39)
	*Cardiovascular (acute)*	AMI risk ↑40% (OR 1.42); highest in first month (OR 2.24); ↑ overall CVD & HF risk (∼2×) (40, 41)
CHRONIC	*Metabolic*	Diabetes mellitus; Weight gain (60%–75%); Dyslipidaemia; Hyperglycaemia (∼5%) (13, 42)
	*Endocrine*	Adrenal insufficiency (37% overall; 44% in OCS-dependent asthma) (43–45)
	*Bone*	Osteoporosis up to 86% (>1 year use); Vertebral fractures 30% after 5–10 yrs; Radiologic osteoporosis 10%–15%, fractures 15%–20% after 5 yrs (13, 14, 42)
	*Neuropsychiatric*	Mood/sleep disorders (40%–60%); anxiety/depression (16%–38% vs. 10%–25% non-users); depression (22%), mania (11%), anxiety (8%), delirium (16%); psychosis (1%–5%, up to 15% high-dose) (13, 22, 46, 47)
	*Ocular*	Cataracts (10%–15%, even at low-dose long-term use) (13, 42)
	*Cardiovascular (chronic)*	Hypertension (30%–35% vs. 25%–29% non-users); ↑ CHD and chronic heart failure (22, 42)
	*Dermatologic*	Skin fragility/easy bruising (30%–40%) (13, 42)

AMI, Acute Myocardial Infarction; CVD, cardiovascular diseases; HF, Heart failure; OCS: oral corticosteroids; CHD, chronic heart disease.

Although ICS are generally considered extremely safe, they might cause both local and systemic AEs. Local AEs commonly associated with ICS are oropharyngeal candidiasis, dysphonia, reflex cough, bronchospasm and pharyngitis. Even if local AEs associated with ICS do not lead to significant morbidity, they diminish compliance, leading to uncontrolled asthma and may reduce patient quality of life (29). ICS systemic AEs (Table 1), observed with high doses is due to the systemic absorption that does not only contribute to efficacy, but might also mediate drug's toxicity (27, 36, 37). Interestingly, even the risk of adrenal insufficiency (AI) has been documented, with estimates suggesting that up to 7% of ICS-only users may be affected (43). Recently, Bloom et al. (24) in a large observational study of two different databases including 162,202 patients followed for 1 year reported a significant association between moderate-to-high ICS doses and acute complications including arrhythmias, pulmonary embolism, and pneumonia emphasizing the importance of adhering to the guidelines' recommendations to use the lowest effective dose of ICS. Interesting, the differences in the relative risk of each complication among different ICS were not significant (1).

### Patients at risk of CS-related adverse events

In clinical practice, CS-related AEs tend to cluster within three principal groups: individuals with substantial cumulative exposure to OCS, patients receiving long-term high-dose ICS, and those with comorbidities or clinical features that heighten susceptibility to systemic steroid toxicity. International recommendations, including GINA, underscore the importance of structured counseling and proactive surveillance in patients on long-term OCS, particularly with respect to bone health and adrenal function (1). Within this framework, screening should be considered a fundamental component of asthma management rather than an intervention reserved solely for the most complex clinical scenarios (42).

Cumulative corticosteroid exposure remains the main determinant of long-term systemic AEs, reflecting not only treatment duration but, more critically, the total dose accrued over time (13, 32, 48). Patients receiving maintenance CS therapy (≥5 mg/day for ≥3 months) constitute a clearly defined high-risk subgroup. Intermittent OCS courses, when recurrent, may also contribute to cumulative toxicity, although generally following a slower trajectory (15). In a recent retrospective study, Casale et al. (42) reported, as expected, an increase risk of CS-related AEs in patients with continuous high OCS exposure (>20 mg/day for 90 days) than in patients using OCS bursts (42). However, even patients with medium OCS exposure (6–12 mg/day) along with patients with 3–4 OCS bursts per year had a significantly greater risk of developing AEs (12, 42). As a general principle, all patients with a cumulative lifetime dose of more than 1 g prednisone equivalent (12) and patients undergoing mOCS tapering should be screened for AEs (1, 49, 50).

Although ICS have a more favorable safety profile than OCS, systemic effects may still arise, particularly at high doses or when using molecules with greater lipophilicity and systemic bioavailability, such as fluticasone propionate (FP) (51).

Finally, screening should extend to patients with pre-existing conditions that could be exacerbated by CS toxicity, as well as to those receiving concomitant CYP3A4 inhibitors, which increase systemic CS concentrations (32, 52–54). Any new-onset signs of CS excess should prompt immediate evaluation, even in the absence of defined exposure thresholds (14).

### How to perform screening of CS-related adverse events

**Figure 1:**
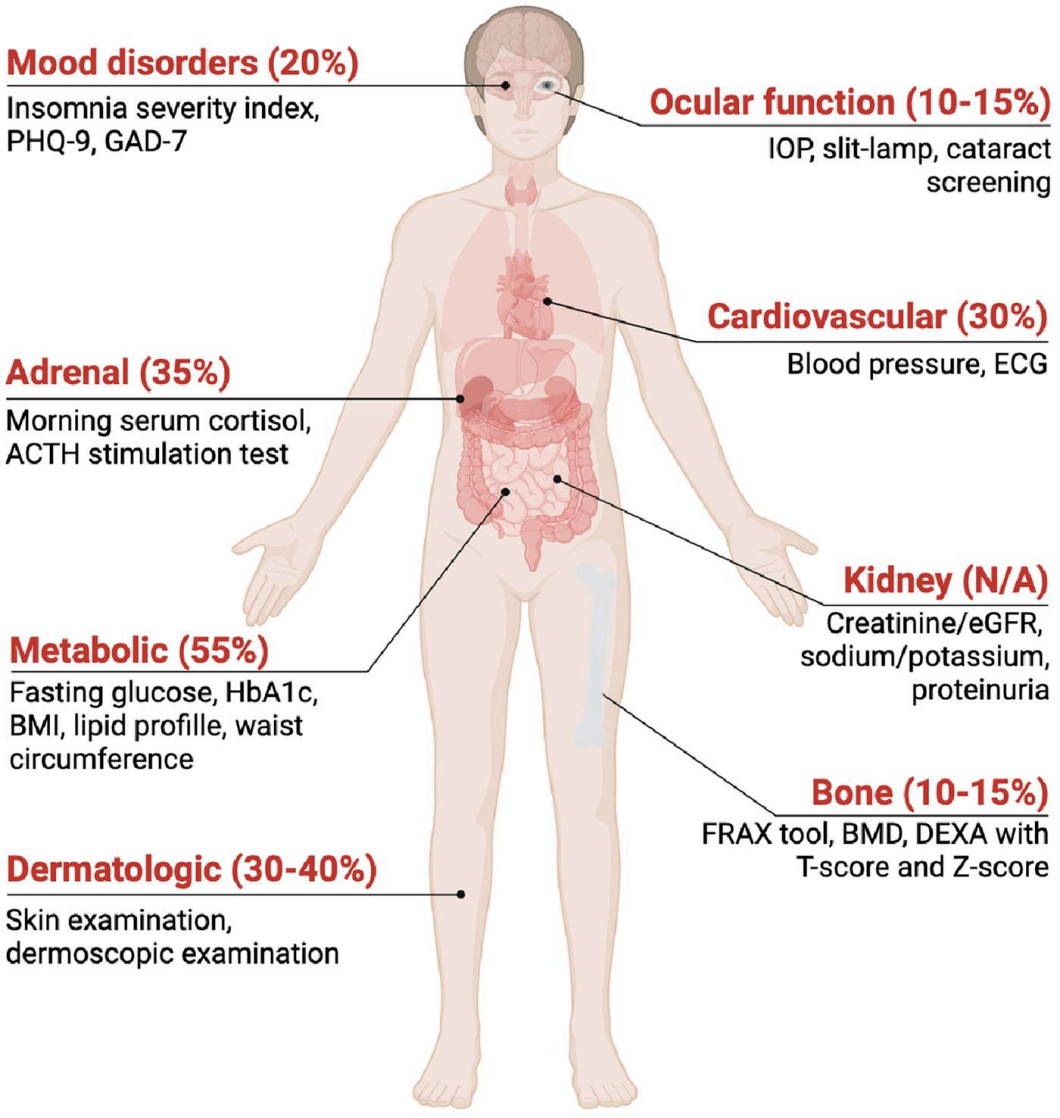
CS adverse events and screeening techniques. PDH-9, Patient Health Questionnaire-9; GAD-7, Generalized Anxiety Disorder-7; IOP, intraocular pressure; ECG, electrocardiogram; HbA1c, glycated hemoglobin; BMI, body mass index; FRAX tool, Fracture Risk Assessment tool; BMD, bone mineral density; DEXA, dual-energy X-ray absorptiometry.

The screening strategy should be proactive rather than reactive and encompass both baseline assessment and longitudinal monitoring. Patients considered at risk of CS-related toxicity (as previously outlined) should be considered for the following screening evaluations (Figure 1):

1. **Metabolic screening**: fasting glucose, postprandial glucose or HbA1c, lipid profile, body mass index (BMI), and waist circumference. CS are known to induce insulin resistance, dyslipidaemia, and central obesity, all of which contribute to increased cardiovascular risk (12, 14). Patients should be educated on symptoms of hyperglycaemia and encouraged to monitor glucose more frequently if symptomatic or diabetic. Once-daily OCS dose in the morning is preferable to reduce glycaemic impact (55).
2. **Adrenal function**: Morning serum cortisol (8:00–9:00 a.m.) is the first-line test to assess hypothalamic–pituitary–adrenal (HPA) axis recovery. Very low values (<100–140 nmol/L) suggest adrenal insufficiency, whereas higher levels (>350–400 nmol/L) indicate adequate adrenal reserve; intermediate results require repeat testing. These thresholds align with major endocrine guidelines (56). When uncertainty persists, the ACTH stimulation test is the gold standard, but not recommended for routine screening in asymptomatic patients (43, 50, 57, 58).
3. **Bone health**: measurement of bone mineral density (BMD) via dual-energy x-ray absorptiometry (DEXA) is recommended in all patients at risk of CS-induced osteoporosis. Fracture risk should be stratified early, ideally within the first 6 months of initiating OCS therapy and reassessed annually. The FRAX tool, a questionnaire for estimating fracture probability, may assist in risk assessment in patients exposed to CS as suggested by international osteoporosis guidelines, such as the National Osteoporosis Guideline Group (NOGG) and the International Osteoporosis Foundation (IOF) (59, 60).
4. **Cardiovascular and renal parameters**: regular monitoring of blood pressure and renal function (creatinine, eGFR, electrolytes) is recommended, since CS might induce hypertension and fluid retention (13). Routine cardiac imaging is not recommended and should be considered only in the presence of clinical indications (61).
5. **Mood disorders**: regular but streamlined screening should be performed. Brief validated questionnaires (e.g., PHQ-9, GAD-7, Insomnia Severity Index) can help detect common neuropsychiatric effects such as depression (22%), anxiety (around 10%), mania or delirium (16%) (46, 62, 63).
6. **Ocular effects**: baseline periodic ocular assessments (intraocular pressure—IOP, slit-lamp exam) are recommended for patients on chronic therapy, particularly those at risk of glaucoma or cataract (64). A recent meta-analysis (65) reported that high-dose ICS (≥1,000 µg/day) and OCS significantly increase the risk of cataract, particularly in patients with asthma or COPD.
7. **Dermatologic manifestations:** a clinical skin assessment every 6 months can identify thinning, bruising, or impaired wound healing, which are often dose- and age-dependent (66). In selected or unclear cases, dermoscopic examination can be considered, particularly in at-risk individuals, to detect early signs of CS-induced skin atrophy (67).

Patient-reported tools, such as the Inhaled Corticosteroid Questionnaire–Short (ICQ-S), may support early identification of ICS-related symptoms in routine clinical practice and facilitate timely management (68).

Monitoring frequency should reflect cumulative CS exposure: in long-term OCS users, metabolic and cardiovascular assessments every 6–12 months and DEXA every 1–2 years are usually recommended (69). Education remains central to prevention and early detection: both clinicians and patients should recognise early signs of CS toxicity.

### How to reduce CS burden

International guidelines emphasise that OCS should be considered the very last option for patients with severe asthma uncontrolled by maximized inhaled therapy (1, 70). If needed, maintenance OCS should only be considered as temporary option and whenever possible a step-down trial is recommended. Protocols for OCS tapering after initiating biologics have been proposed since 2021 by Suehs and co-authors and more recently in the PONENTE and WAYFINDER studies (49, 50, 71).

For patients with severe asthma, current available biologics are anti-IgE (omalizumab), anti-IL(-5 (mepolizumab), anti-IL-5R*α* (benralizumab), anti-IL-4R*α* (dupilumab) and anti-TSLP (thymic stromal lymphopoietin, tezepelumab) as add-on treatment (1, 21). All biologics have been shown to reduce severe exacerbations and OCS burden in different selected population of patients with severe asthma (50, 72–75). Although only up to 30% of patients achieves clinical remission with the use of biologics (76), whereas a clinical response, defined as a reduction of exacerbations and OCS use is much more frequent (77). Interesting, studies in real life reported even better results with a reduction of OCS in more than 80% of patients (78–80) and a reduction of ICS in about 30% (79). Nevertheless, it is important to emphasize that, as far as we know, the biological drugs currently available do not necessarily have broad spectrum of anti-inflammatory activity that steroids have, as recently demonstrated by Howell (81) showing an additive anti-inflammatory effects on the top of mepolizumab. These effects might be responsible of residual exacerbations observed in patients treated with mepolizumab for severe eosinophilic asthma. This finding might also explain why inhaled corticosteroids still play a role even in patients treated with biologics as reported in the SHAMAL study suggesting that ICS reduction may be attempted only in carefully selected and adequately controlled patients, while complete withdrawal cannot currently be considered a safe strategy (82). On the other hand, as even high dose ICS may induce AEs, GINA 2025 guidelines, in order to reduce burden of CS, suggest to adopt the Track 1 strategy, as it has been shown to reduce risk of severe exacerbations and OCS exposure compared to SABA-based regimens (83, 84) and if needed to limit high doses ICS/LABA to 3–6 months. Moreover, before increasing ICS dose, especially in patients with T2-low asthma, others effective add-on medication should be considered. Indeed, it has been shown that the concomitant administration of long-acting muscarinic antagonist (LAMA) with ICS/LABA improves both lung function and asthma control (85), and the use of a single-inhaler for triple therapy (SITT) may improve patient's adherence, maintaining control with low doses of ICS (86, 87).

Despite major advances in asthma management, exacerbations remain a key challenge, as they continue to require systemic corticosteroids as first-line treatment (1). Although biologics have shown potential benefits, their use in the acute setting requires further investigation (88–92). Notably, exacerbation phenotyping may represent a valuable strategy to provide targeted treatment, avoiding inappropriate CS use (93). Indeed, the recent PRISMA study suggests that objective clinical improvement following OCS therapy is largely confined to T2-high exacerbations (94). Conversely, in patients receiving biologic therapy, exacerbations may be less responsive to oral corticosteroids, particularly when triggered by infections rather than eosinophilic inflammation (81). Finally, management of patients who fail to respond to biologics remains challenging; potential strategies include switching between biologics (95, 96) and long-term azithromycin, which reduces moderate-to-severe exacerbations in both T2-high and T2-low asthma (97, 98).

## Discussion

In patients with asthma the overuse of systemic CS is still too common despite their well-documented risk of AEs. While the risk related to maintenance OCS is well established, the risk associated with the use of short OCS bursts has not yet been fully recognised probably due to the absence of a well-defined threshold for OCS-related AEs. OCS stewardship among both specialists and general practitioners could be a valid option to reduce the inappropriate use of OCS. In clinical practice, patients are often referred to a severe asthma specialist after several courses of OCS, despite all international guidelines consider asthma as uncontrolled after only two OCS courses (1, 70). This delay precludes the opportunity to optimize asthma therapy and to initiate biologics early, increasing the risk of CS-related AEs. Furthermore, severe asthma centres should incorporate screening for CS-related AEs for all patients at risk, particularly for those who have received a cumulative dose greater than 1 g of prednisone equivalent over the course of their lifetime (99). As it's known that even high dose ICS are no longer considered risk-free, inhaled therapy should always be re-evaluated in clinical practice and reduced to the lowest effective dose possibly using them in combination with long acting bronchodilators (87). In particular, as recent evidence suggests the tapering of ICS in patients who achieve clinical remission under biologic therapy is achievable (82), a proper algorithm for ICS tapering should be proposed to improve the management of patients with severe asthma. Finally, an opportunity to reduce the steroid burden would be to offer biologic therapy to patients with moderate asthma uncontrolled by medium doses of ICS/LABA (100).

In conclusion, to date corticosteroids are still overused and clinicians are not fully aware of their impact on health. Stewardship on CS use, both systemic and inhaled, is needed to reduce adverse events and to promote screening and alternative treatment, including the use of the lowest effective ICS dose.
